# Disturbance amplifies sensitivity of dryland productivity to precipitation variability

**DOI:** 10.1126/sciadv.adm9732

**Published:** 2024-07-26

**Authors:** Tyson J. Terry, Osvaldo E. Sala, Scott Ferrenberg, Sasha C. Reed, Brooke Osborne, Samuel Jordan, Steven Lee, Peter B. Adler

**Affiliations:** ^1^ Department of Wildland Resources and the Ecology Center, Utah State University, Logan, UT 84322, USA.; ^2^ School of Life Sciences, Arizona State University, Tempe, AZ 85287, USA.; ^3^ Department of Ecosystem and Conservation Sciences, University of Montana, Missoula, MT 59812, USA.; ^4^ U.S. Geological Survey, Southwest Biological Science Center, Moab, UT 84532, USA.; ^5^ Department of Environment and Society, Utah State University, Moab, UT 84532, USA.; ^6^ U.S. Geological Survey, Western Ecological Research Center, Wawona, CA 95389, USA.

## Abstract

Variability of the terrestrial global carbon sink is largely determined by the response of dryland productivity to annual precipitation. Despite extensive disturbance in drylands, how disturbance alters productivity-precipitation relationships remains poorly understood. Using remote-sensing to pair more than 5600 km of natural gas pipeline corridors with neighboring undisturbed areas in North American drylands, we found that disturbance reduced average annual production 6 to 29% and caused up to a fivefold increase in the sensitivity of net primary productivity (NPP) to interannual variation in precipitation. Disturbance impacts were larger and longer-lasting at locations with higher precipitation (>450 mm mean annual precipitation). Disturbance effects on NPP dynamics were mostly explained by shifts from woody to herbaceous vegetation. Severe disturbance will amplify effects of increasing precipitation variability on NPP in drylands.

## Teaser

Disturbance causes shifts in plant composition that amplify interannual variability of dryland plant productivity.

## INTRODUCTION

Arid and semi-arid ecosystems (drylands) play a dominant role in the interannual variability and long-term trend of the terrestrial global carbon sink (*1*, *2*). Interannual variability of net primary productivity (NPP) in drylands is driven by a high sensitivity of NPP to variability in annual precipitation. In contrast, in ecosystems with higher precipitation, production is less limited by water availability than by mineral nutrients and light (*3*, *4*). Plant community structure modifies NPP-precipitation dynamics through the influence of functional traits representing acquisitive versus conservative growth strategies (*5*–*7*). Physical disturbance is likely to modify the relationship between NPP and precipitation through direct and marked effects on plant functional composition and resource availability (*8*), but these potential interactions remain poorly understood. Closing this knowledge gap is critical, as most drylands are simultaneously experiencing widespread physical disturbance (*9*–*11*) and increasing precipitation variability (*12*, *13*).

Dryland systems have long been affected by grazing (*14*) and resource extraction (*15*) and are now increasingly exposed to novel disturbances such as accelerated wildfire regimes (*16*) and spatially extensive infrastructure tied to extractive and renewable energy (*9*, *17*). Disturbance modifies the plant community by either initiating secondary succession, typically replacing mature, long-lived vegetation with early successional species, or selecting for species with resilience to the specific disturbance pressure, as when heavy grazing leads to shrub dominance (*14*). In drylands, severe physical disturbance often replaces long-lived shrubs and trees with herbaceous species (*18*, *19*) with shallower root systems, large seedbanks (annuals), or high meristem densities (perennials) that not only enable quick growth during years of high precipitation, but also increase susceptibility of aboveground productivity to drought (*6*). Despite our understanding of the life-history traits of early successional species (*20*), the impact of disturbance-mediated shifts in plant species composition on precipitation-production relationships remains unknown.

Comparing postdisturbance productivity and its relationship with precipitation across regional climate gradients is difficult due to the idiosyncrasies of natural and anthropogenic disturbances that vary in type, intensity, size, and timing. Anthropogenic disturbances such as pipeline corridors provide an opportunity to study ecosystem dynamics following a spatially consistent physical disturbance involving vegetation removal and disruption of the surface soil. Here, we use 34 years of remote sensing data across 5600+ km of natural gas pipeline corridors and adjacent undisturbed vegetation to study the effect of a uniform pulse disturbance on productivity across broad precipitation gradients in North American drylands. We asked (i) how does physical disturbance affect average NPP and the sensitivity of annual NPP to interannual variation in precipitation? and (ii) are disturbance effects on NPP explained by shifts in the abundance of plant functional groups? We hypothesized that disturbance would decrease average NPP and increase the sensitivity of NPP to annual anomalies in precipitation due to shifts from long-lived woody plants to short-lived herbaceous species with accelerated growth strategies. We predicted that effects of disturbance would be strongest in locations with low average precipitation, where replacement of shrubs with herbaceous cover may exacerbate water limitations on NPP. This prediction would validate previous results that indicate that vegetation structure modifies the sensitivity of aboveground primary production to interannual precipitation variability (*5*, *6*).

We analyzed average annual NPP across gradients of mean annual precipitation (MAP) traversed by multiple pipelines and the sensitivity of NPP to interannual variation in precipitation (*3*) for individual pixels within and adjacent to each pipeline corridor (*3*, *5*, *6*). We defined the temporal sensitivity of NPP to precipitation at a given location as the slope of the linear relationship between NPP (g C m^−2^ year^−1^) and annual precipitation (mm year^−1^). Thus, an increase in the temporal sensitivity indicates a greater increase of annual production in a year of above-average precipitation or a greater decrease in a year of below-average precipitation. We did not consider nonlinear relationships and leveraged linear interaction terms to understand relationships. To address our first research question, we used a linear model (disturbance-only model, Table 1) to quantify effects of disturbance on average production and temporal sensitivity of pixels within disturbed pipeline corridors and in undisturbed, adjacent, comparison pixels. This model uses interactions between years since disturbance (YSD), MAP, and annual deviations from that mean (Pdev) to understand disturbance effects on average productivity and sensitivity (Table 1). This and subsequent models include a random effect of pipeline identity to account for differences in corridor width and construction impacts between individual pipelines.

**Table 1.** Model selection table with values indicating overall model fit and fixed-effect covariates of linear models used. The disturbance model includes covariates of years since disturbance (YSD) and precipitation data of mean annual precipitation (MAP) and annual deviations from MAP (Pdev). The composition model includes covariates of woody (Woody) and herbaceous (Herb) plant cover and precipitation data. The composition + disturbance model includes woody and herbaceous plant cover, precipitation data, and YSD.

Model	df	*R* ^2^	*R*^2^ without random effects	Delta AICc	Weight	Covariates
Composition + Disturbance	13	0.624	0.619	10.6	0.005	MAP, Pdev, MAP*
						Pdev, MAP*Woody, MAP*
						Herb, Pdev*Woody,
						Pdev*Herb,
						MAP*Woody*1/√YSD,
						MAP*Herb*1/√YSD,
						Pdev*Woody*1/√YSD,
						Pdev*Herb*1/√YSD
Composition	9	0.623	0.621	0	0.995	MAP, Pdev, MAP*Pdev,
						MAP*Woody, MAP*Herb,
						Pdev*Woody, Pdev*Herb
Disturbance	8	0.604	0.477	13,037	0	MAP, Pdev, MAP*Pdev
						MAP*1/√YSD,
						MAP*Pdev*1/√YSD

## RESULTS AND DISCUSSION

**Fig. 1.**
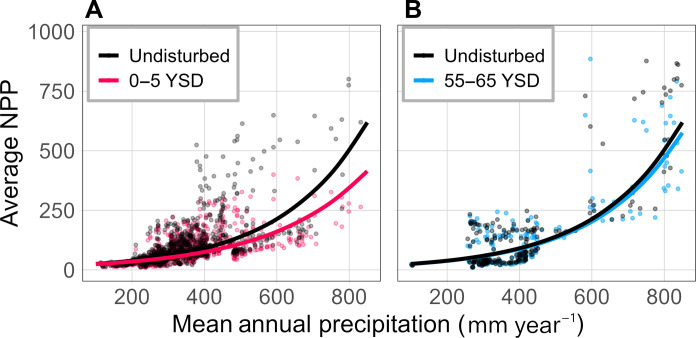
Effects of disturbance on average net primary production (NPP, g C m^2^ year^−1^) across a spatial gradient of mean annual precipitation (MAP, mm year^−1^). Effects of disturbance are shown 0 to 5 years (**A**) and 55 to 65 years (**B**) since disturbance (YSD). Colored points show raw values of mean NPP for subsets of the data based on time since disturbance, while lines represent predictions from a model fit to the full dataset (conditional *R*^2^ = 0.60) assuming annual precipitation matches MAP. Black points represent mean values from observations of adjacent undisturbed vegetation during the same time frame.

**Fig. 2.**
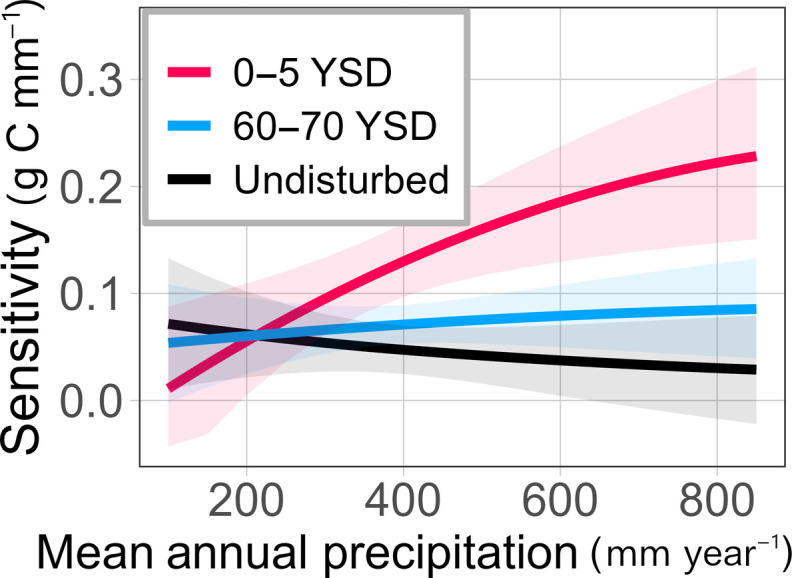
Effects of disturbance on the temporal sensitivity of NPP to interannual variation in precipitation increase across a gradient of MAP (mm year^−1^). Lines show the predicted temporal sensitivity (change in annual production per change in annual precipitation) at different years since severe disturbance (YSD). Line predictions come from a linear model trained with the full dataset (conditional *R*^2^ = 0.60). Colored margins represent 95% confidence intervals for model predictions.

We found that initial effects of pipeline disturbance decreased average NPP by 6 to 29% (term = MAP*YSD, *t* = −5.47, *P* < 0.001; Fig. 1A) and increased the temporal sensitivity of NPP to annual precipitation up to fivefold (term = Pdev*YSD, *t* = 4.18, *P* < 0.001; Fig. 2). Reductions in average NPP and increases in sensitivity were both larger and longer-lasting at high precipitation locations (MAP >450 mm) where average production was predicted to be 4 to 7% lower and twice as sensitive to annual precipitation than undisturbed controls even after 55 to 65 years of recovery following pipeline construction (Figs. 1B and 2). Impacts were smaller in locations with MAP <300 mm, where average productivity and temporal sensitivity were largely unaffected (Figs. 1 and 2).

In the absence of disturbance, the sensitivity of NPP to interannual variation in precipitation was slightly greater in water-limited environments and decreased with increases in MAP (Fig. 2, black line). This pattern, though weak in our data, is consistent with previous work (*4*) and may reflect increasing limitation of productivity by nonwater resources (e.g., temperature, light, carbon, and nutrients) in ecosystems with higher MAP (*1*). Disturbance reversed this expected pattern: in the years following pipeline construction, the sensitivity of NPP to annual precipitation was highest in locations with high MAP (Fig. 2, red line). Disturbance can change resource availability and use by releasing nutrients stored in biomass and altering the pool of plant functional traits that determine resource uptake and precipitation-use efficiency (*4*, *12*). We hypothesize that impacts of disturbance on sensitivity were disproportionately large in locations where nonwater resources (e.g., carbon and nutrients) limit undisturbed production and, following a disturbance, a pulse of previously limited mineral resources then makes water a more primary limiting factor. The pulse of nutrients, such as phosphorus or nitrogen, may come from a release of resources (*8*) formerly stored in slow turnover mineral-associated organic matter that was enhanced due to changes in the soil climate (*21*). A meta-analysis showing that responses to N fertilization increase with MAP supports this hypothesis (*22*). While a change in resource limitation is one mechanism that could explain the disproportionately large effects of disturbance at higher MAP, we could not test this hypothesis, and we highlight the need for future studies to investigate how nonwater resource limitation shifts following disturbance.

To address our second research question, about the role of species composition in mediating disturbance impacts, we compared three linear regression models. The first model (disturbance model, Table 1) used only YSD and precipitation covariates (MAP and Pdev) to capture changes in production and its sensitivity. The second model (composition model, Table 1) included no explicit disturbance covariates, and instead relied on interactions between precipitation and woody (Woody) and herbaceous plant cover (Herb). The third model (disturbance + composition model) included both plant composition, YSD, and precipitation covariates (Table 1), with interactions that allow average production and temporal sensitivity of different functional groups to change with time since disturbance.

**Fig. 3.**
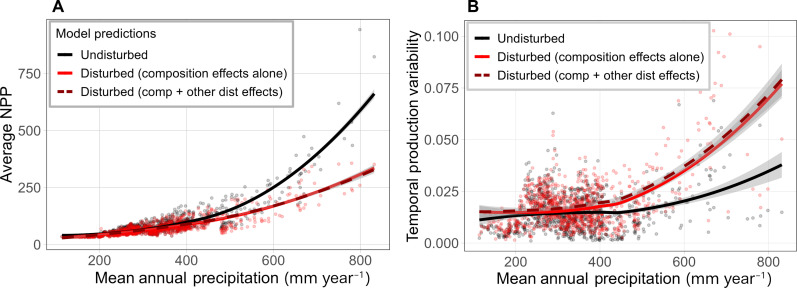
NPP values at disturbed sites and their undisturbed controls 0 to 10 years after disturbance across a gradient of MAP. (**A**) shows predicted average NPP for each pixel and (**B**) shows predicted temporal variance (coefficient of variation) of NPP. Black points and lines represent observations and model predictions, respectively, for undisturbed control pixels. Red points and lines represent observations and model predictions for disturbed pixels. The red solid line represents model predictions for the composition-only model, in which disturbance can only affect production by changing composition. The red dashed line represents model predictions for the composition + disturbance model, in which disturbance can affect both composition and the average productivity and sensitivity of functional plant groups. Polygons surrounding lines represent 95% confidence intervals for model predictions.

**Fig. 4.**
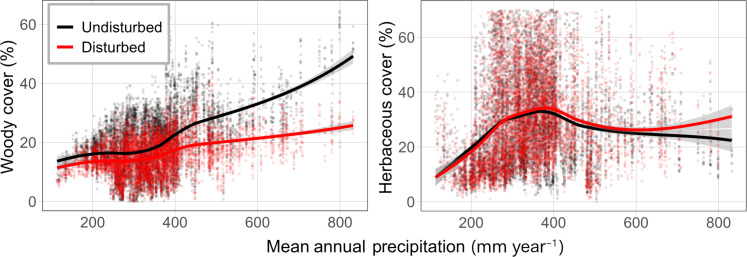
Plant functional type cover in disturbed pixels (red) and their respective undisturbed controls (black). Disturbed points represent annual cover values for the first 10 years following disturbance; undisturbed points represent the annual cover values of undisturbed controls for the same sites and years as the disturbed data. Smoothed lines are representing general patterns of the data (with shaded margins indicating 95% confidence intervals of smooth prediction lines) for disturbed (red) and undisturbed (black) cover values. For display purposes only, values of herbaceous cover >70% are not shown. The *R*^2^ values for the left and right plots are 0.26 and 0.13, respectively.

The model based solely on changes in plant functional composition, ignoring disturbance history, explained more variation in annual NPP than a model informed only by time since disturbance (*R*^2^ = 0.62 versus 0.60), and explained as much variation in the data but with a lower Akaike information criterion (AIC) compared to the model that was informed by both time since disturbance and composition (*R*^2^ = 0.62) (Table 1). The fact that the model allowing the production sensitivities of each functional group to change with disturbance did not improve model fit indicates that changes in woody and herbaceous plant cover were responsible for most disturbance effects on production. Moreover, the proportion of variance explained by the random effects of individual pipeline identities diminished to near zero when models included plant functional cover (Table 1), suggesting that differences between individual pipeline disturbances and their subsequent impacts on production can be mostly explained by their respective effects on plant functional group composition. To visually compare impacts of altered plant functional composition, we applied the two models with composition covariates to a subset of disturbed and undisturbed pixels for which we have cover data from 0 to 10 years following disturbance and found that the pattern of increasing disturbance effects with MAP could be largely attributed to shifts in plant functional type cover (Fig. 3). Locations with the greatest decrease in average productivity and largest increase in temporal sensitivity were also in locations that lost the most woody plant cover while maintaining or potentially gaining herbaceous plant cover following severe disturbance (Fig. 4).

The model that allowed the productivity of functional groups to vary with time since disturbance showed increases in both average productivity (term = Herb*MAP*YSD, *t* = 9.087, *P* < 0.001) and temporal sensitivity (term = Herb*Pdev*YSD, *t* = 2.421, *P* = 0.015) of herbaceous cover after disturbance, and decreases in both average productivity (term = Woody*MAP*YSD, *t* = −7.431, *P* < 0.001) and temporal sensitivity (term = Woody*Pdev*YSD, *t* = −2.833, *P* = 0.005) of woody cover after disturbance. We speculate that these changes are due to two potential processes: First, postdisturbance increases in soil water (*23*) and nutrients (*24*) may provide competitive release for herbaceous species that are often outcompeted by woody species under conditions of low resource availability (*25*). Second, species turnover following disturbance may be greater within the herbaceous functional group, where shifts from perennial herbaceous species to invasive annual herbaceous species following disturbance are common in drylands (*19*, *26*).

We found that changes in plant composition specifically shifts from woody to herbaceous vegetation and increased temporal variability of NPP across a gradient of mean precipitation more than shifts in mean precipitation gradient alone (Fig. 3B). This may indicate that while patterns of precipitation variability and plant composition that co-occur across a mean precipitation gradient both influence production variability (*6*, *27*, *28*), plant composition may be the primary determinant of production variability.

The larger impacts of disturbance in locations with higher MAP that we documented may also be linked to regional patterns of degradation. Dry locations with poor soil development have historically been unfit for agricultural use and thus have been subjected to intense historical grazing pressure (*14*). Our approach uses current vegetation outside of disturbed pipeline corridors as “undisturbed” data and does not account for deviations between current and potential vegetation, or how those deviations may correlate with regional precipitation gradients. Our approach could underestimate impacts of disturbance in dry locations if they are in fact more degraded from historical land use.

Our results indicate that disturbance will likely amplify impacts of precipitation variability on production and carbon cycling within dryland systems. Our data show 10 to 100% increases in interannual variability of annual NPP in locations that receive more than 400-mm annual precipitation (Fig. 3B). This is particularly concerning given the current patterns of increasing precipitation variability in drylands worldwide (*12*, *29*, *30*). Greater frequency or extent of disturbances would further increase the large influence drylands have on the interannual variability of the global carbon cycle (*1*, *2*). Higher interannual variability in production may also cause reduction of habitat and microclimate refugia provided by stable plant communities during years of dry conditions.

## MATERIALS AND METHODS

### Pipelines

Our analysis utilized four natural gas pipeline corridors within the western United States: Kern River Pipeline (2702 km, built in 1992), Ruby Pipeline (1090 km, built in 2011), El Paso Natural Gas Pipeline (1040 km, built in 1946), and Northwestern Pipeline (860 km, built in 1960) (fig. S1). We selected these pipelines based on width (~30 m), to allow use of fine-scale remote sensing products within the disturbed pipeline corridors, and length (>160 km), to capture disturbance effects along precipitation gradients within and across unique dryland systems. We used a high-resolution satellite basemap (<1 m resolution) in Google Earth Engine (*31*) and pipeline maps from the National Pipeline Mapping System (*32*) to visually draw a reference line in the center of each pipeline corridor and create a parallel reference line of undisturbed vegetation of similar topography and land use adjacent to the pipeline corridor (within 60 to 150 m). Only pipeline pixels whose centroid fell within 3 m of the pipeline reference line and their respective closest undisturbed neighbor pixel along the undisturbed reference line were included in the dataset (fig. S2). We also excluded urban and agricultural locations from the dataset. NPP pixels within the pipeline corridor and the undisturbed reference line (30 m) were respectively averaged to the coarser spatial scale of precipitation pixels (1000 m) to study precipitation effects on NPP (fig. S2). Our analysis consists mostly of pixels that have been classified as drylands, meaning that the ratio of precipitation to potential evaporation in these locations is less than 0.7. Our analysis does include a small portion of pixels that exceed this value (fig. S1), but they were retained in the dataset as they belong to small breaks from “drylands” that occur in shallow mountain ranges that are surrounded by drylands and also to extend the observed precipitation gradient to the edge of dryland pixels.

Pipeline construction involves the removal of all vegetation within the corridor via heavy equipment and results in churning of the surface soil. After topsoil is pushed aside and subsequently redistributed following pipeline installation, pipeline corridors are drill seeded with a restoration seed mix that is region-specific and typically chosen by a federal agency within each geographic district. Seed mixes include native shrub, forb, and perennial grass species with composition varying according to undisturbed vegetation type (e.g., desert scrub, sagebrush scrub, piñon juniper woodland, and desert grassland). To our knowledge, there have been no large-scale secondary disturbances or ecological treatments on the four pipelines included in this study following the initial pipeline construction. Although pipeline construction is not a close analog of common natural disturbances, it provides a near-uniform physical disturbance across space, allowing us to ask questions regarding effects of disturbance across abiotic gradients. Moreover, pipeline construction is similar to other prevalent anthropogenic physical disturbances on plants and soils, such as energy infrastructure and frequent vehicle traffic, which occur in all of the ecosystems included in our study (*9*, *11*). Using pipeline construction as a natural experiment allowed us to compare impacts of a replicated disturbance on precipitation-production relationships across the region, a comparison that would not be possible due to variation in the type, frequency, and severity of natural disturbances at these scales.

### Data

We used 34 years (1986 of 2019) of remotely sensed estimates of annual NPP (*33*) to identify spatial and temporal trends of production following pipeline construction. This NPP dataset uses a modified version of the MOD17 algorithm to improve accuracy and allow use with Landsat imagery at a 30-m spatial resolution (*33*). The approach uses reflectance values of photosynthetic active wavelengths as well as meteorological inputs (short wave radiation, daily minimum and maximum temperature, and vapor pressure deficit) to calculate light use efficiency and scale rates of respiration. Calculations account for inter-annual shifts of plant composition within pixels (*33*) by shifting maintenance respiration with changes in vegetation (*33*), allowing production-reflectance relationships to shift with novel plant composition following disturbance. Estimates of undisturbed production and its sensitivity to annual precipitation based on this dataset are similar to those based on less-processed proxies for productivity, such as normalized difference vegetation index and enhanced vegetation index (*4*).

We also used a fractional plant cover dataset (*34*), generated from a convoluted neural network model that uses reflectance values to provide annual cover estimates for tree, shrub, perennial herbaceous, and annual herbaceous plant functional groups at a 30-m spatial resolution. We simplified these groups into woody (shrub + tree) and herbaceous cover (annual + perennial) to facilitate interpretation and reduce the number of parameters in our models.

We obtained precipitation data from the gridded climate product Daymet (*35*), with MAP calculated as average total precipitation for each water year (October 1 to September 30) during the years of analysis (1986 to 2019), and annual deviations in precipitation calculated as the difference between total precipitation of each water year and the MAP for that pixel.

We excluded barren locations with average or interannual production values of <10 g*C*year/m^2^ due to low confidence in our dataset to detect meaningful interannual variation in NPP in locations with very sparse vegetation and a lot of bare ground. We also excluded pixels from both NPP and fractional cover datasets with high levels of temporal or spatial smoothing by only using pixels with 80% cloud-free imagery during the growing season. We excluded data from the initial year of the pipeline construction due to complications that arise from variable pipeline construction timing and its subsequent influence on annual NPP.

### Modeling disturbance effects on average production and sensitivity

To answer our first research question, we used a mixed effect linear model implemented in the R package lme4 (*36*, *37*) to analyze disturbance effects on NPP across a gradient of mean precipitation and temporal sensitivity of NPP to interannual variation in precipitation (Eq. 2). This disturbance model included main effects of MAP, annual deviations in precipitation (Pdev) around the MAP at each location, and their interaction. This interaction (β_3_) term allowed sensitivity to change across space. We incorporated disturbance as a covariate (YSD) to allow disturbance to affect NPP relationships with MAP, Pdev, and their interaction. The YSD metric was calculated as 1 divided by the square root of years since initial disturbance and was chosen to allow for large initial impacts of disturbance that dissipate toward zero over time (Eq. 1). All undisturbed data points were given a disturbance covariate of 0 to cancel disturbance effect coefficients (Eq. 1), allowing us to fit both undisturbed and disturbed data within the same model. Subscripts indicate parameters that vary across years (*t*) and space (*x*). We also incorporated a random term for pipeline identity (σ*_x_*) interacting with our YSD metric to account for differences among pipelines in corridor width, initial construction, and restoration efforts. Significant (*P* < 0.05) interaction terms involving disturbance indicate that disturbance is changing average production (β_4,6_) and/or sensitivity (β_5,6_). Interpretation of coefficients is provided in table S1.

$${\text{YSD}}_{t}=\left\{ \begin{array}{c} \frac{1}{\sqrt{{\text{YSD}}_{t}}},\;\text{YSD}>0\\ 0,\;\text{if undisturbed} \end{array}\right.$$ (1)

(Disturbance model)

$$\begin{array}{c} \text{Ln}\left({\text{NPP}}_{x,t}\right)=\alpha +\beta_{1}{\text{MAP}}_{x}+\beta_{2}*{\text{Pdev}}_{t}+\beta_{3}*{\text{MAP}}_{x}*{\text{Pdev}}_{t}+\\ \beta_{4}*{\text{MAP}}_{x}*{\text{YSD}}_{t}+\beta_{5}*{{\text{Pdev}}_{t}}^{*}{\text{YSD}}_{t}+\beta_{6}*{\text{MAP}}_{x}*{\text{Pdev}}_{t}*{\text{YSD}}_{t}+\\ \sigma_{p}*{\text{YSD}}_{t}+\varepsilon_{x} \end{array}$$ (2)

### Model comparison

To answer our second research question, we compared the fit of two additional linear models with our disturbance model to quantify how changes in functional composition drive postdisturbance production dynamics. We did this by creating a composition model (Eq. 3) that predicts NPP across space and time using solely functional type cover and precipitation covariates, ignoring information about disturbance history, and compared the fit against another model, our composition + disturbance model (Eq. 4), that added interaction terms to allow disturbance to change average productivity and sensitivity of each plant functional type. Both Eqs. 3 and 4 also contain a random effect of pipeline identity (σ). We then compared all models using the corrected Akaike information criterion (AICc) and coefficient of determination *R*^2^ to quantify the portion of disturbance effects caused by changes in functional composition and effects of disturbance on average productivity and sensitivity of functional groups.

Our composition model (Eq. 3) excluded disturbance effects beyond changes in functional composition and models the main effects of MAP, Pdev, and their interaction with woody (Woody) and herbaceous (Herb) plant cover (Eq. 3). Our composition + disturbance model (Eq. 4) was identical to the prior model (Eq. 3) but included additional interaction terms (β_7 − 10_) that allowed a disturbance metric (Eq. 1) to influence relationships between NPP and MAP and Pdev for each (woody or herbaceous) functional group. Within the composition + disturbance model, significant (*P* < 0.05) interaction terms including disturbance indicate that disturbance is changing average productivity (β_7,8_) and/or sensitivity (β_9,10_) of a functional group.

(Composition model)

$$\begin{array}{c} \text{Ln}\left({\text{NPP}}_{x,t}\right)=\alpha +\beta_{1}*{\text{MAP}}_{x}+\beta_{2}*{\text{Pdev}}_{t}+\beta_{3}*{\text{Herb}}_{t}*{\text{Pdev}}_{t}+\\ \beta_{4}*{\text{Woody}}_{t}*{\text{Pdev}}_{t}+\beta_{5}*{\text{Herb}}_{t}*{\text{MAP}}_{x}+\beta_{6}*{\text{Woody}}_{t}*{\text{MAP}}_{x}+\\ \sigma_{p}+\varepsilon \end{array}$$ (3)

(Composition + disturbance model)

$$\begin{array}{cc} \text{Ln}\left({\text{NPP}}_{x,t}\right)= & \alpha +\beta_{1}*{\text{MAP}}_{x}+\beta_{2}*{\text{Pdev}}_{t}+\beta_{3}*{\text{Herb}}_{t}*{\text{Pdev}}_{t}+\\ & \beta_{4}*{\text{Woody}}_{t}*{\text{Pdev}}_{t}+\beta_{5}*{\text{Herb}}_{t}*{\text{MAP}}_{x}+\\ & \beta_{6}*{\text{Woody}}_{t}*{\text{MAP}}_{x}+\beta_{7}*{\text{Herb}}_{t}*{\text{MAP}}_{x}*{\text{YSD}}_{t}+\\ & \beta_{8}*{\text{Woody}}_{t}*{\text{MAP}}_{x}*{\text{YSD}}_{t}+\beta_{9}*{\text{Herb}}_{t}*{\text{Pdev}}_{t}*{\text{YSD}}_{t}+\\ & \beta_{10}*{\text{Woody}}_{t}*{\text{Pdev}}_{t}*{\text{YSD}}_{t}+\sigma_{p}+\varepsilon \end{array}$$ (4)

### Model checks

We assessed diagnostic plots of each model (figs. S3 to S5) to ensure that assumptions of normality and heteroscedasticity were met. To ensure that spatial autocorrelation was not driving patterns in our model, we constructed a semivariogram to determine the threshold distance between points at which model residuals were no longer spatially correlated (range) and then created a distance matrix between all the sites to determine the proportion of the data under the range value that was spatially autocorrelated (fig. S6). We found that only 6% of our residuals were spatially autocorrelated for the disturbance-only model, which had the poorest fit to the data of the three models (fig. S7). We also completed this process for both the composition-only and composition + disturbance models, both of which had lower values of autocorrelation of residuals than the disturbance-only model (fig. S7). To ensure there was no collinearity within our model parameters, we analyzed the variable inflation factor following model fit and found that all variables had variance inflation factors of less than 3.

## Supplementary Materials

### This PDF file includes:

Figs. S1 to S7 Table S1 References
