# Supplementary material for: Disturbance amplifies sensitivity of dryland productivity to precipitation variability
Source: Sci Adv. 2024 Jul 26;10(30):eadm9732. doi: 10.1126/sciadv.adm9732 (PMC11277371; doi:10.1126/sciadv.adm9732)
Supplement: Supplementary file 1 — Figs. S1 to S7 Table S1 References [file sciadv.adm9732_sm.pdf]

Science Advances
AAAS

Supplementary Materials for

Disturbance amplifies sensitivity of dryland productivity to precipitation variability

Tyson J. Terry *et al.*

Corresponding author: Tyson J. Terry, tysonjterry@gmail.com

Sci. Adv. **10**, eadm9732 (2024)
DOI: 10.1126/sciadv.adm9732

This PDF file includes:

Figs. S1 to S7
Table S1
References

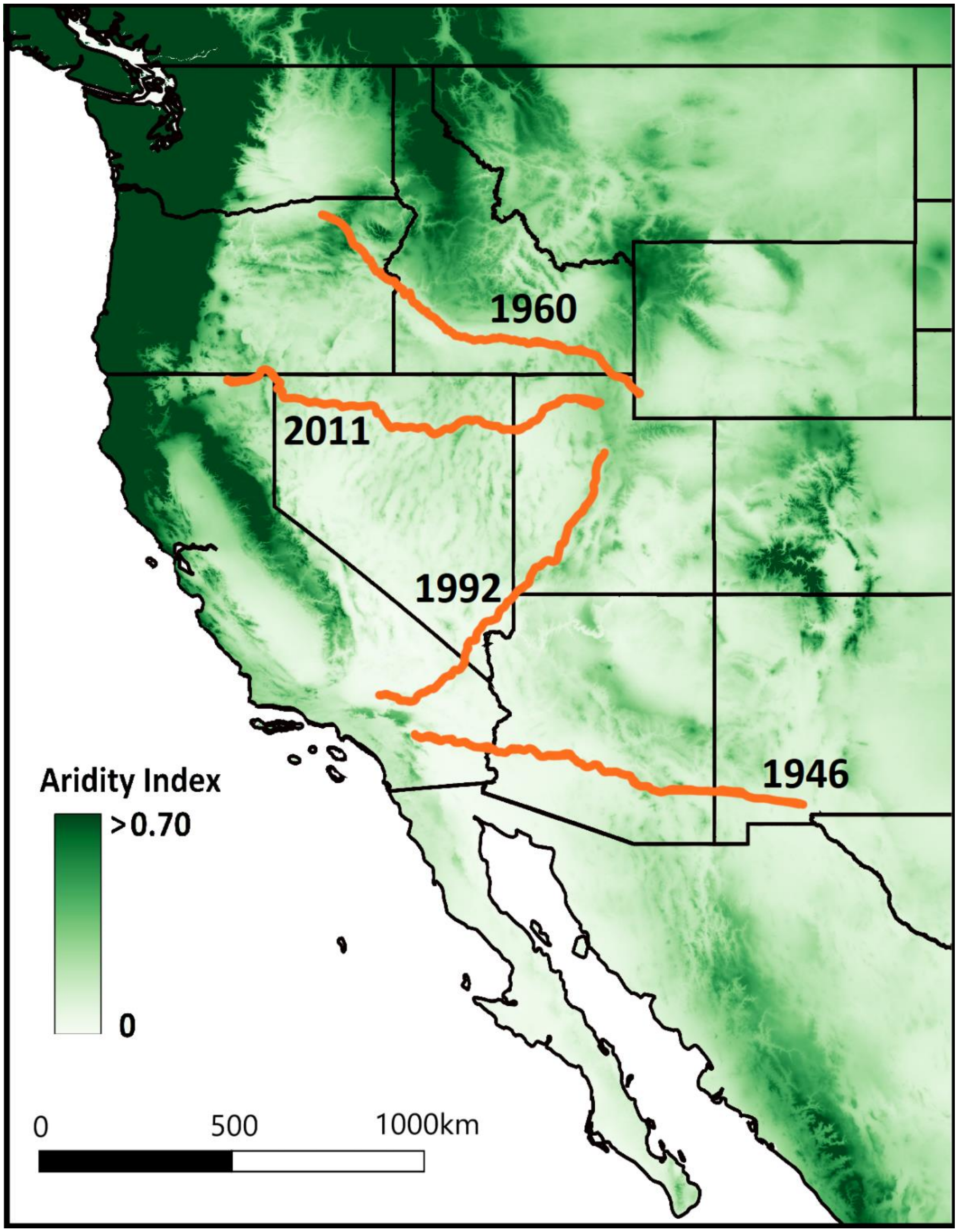

Fig. S1.
Map of the Western United States indicating the location of the four pipelines used in this study along with the years their construction was completed. The color indicates aridity index (Aridity Index = MAP/ MAET where MAP = Mean Annual Precipitation and MAET = Mean Annual Reference Evapotranspiration). Locations with an aridity index value less than 0.7 are generally considered drylands. Index was obtained from a previous study (38).

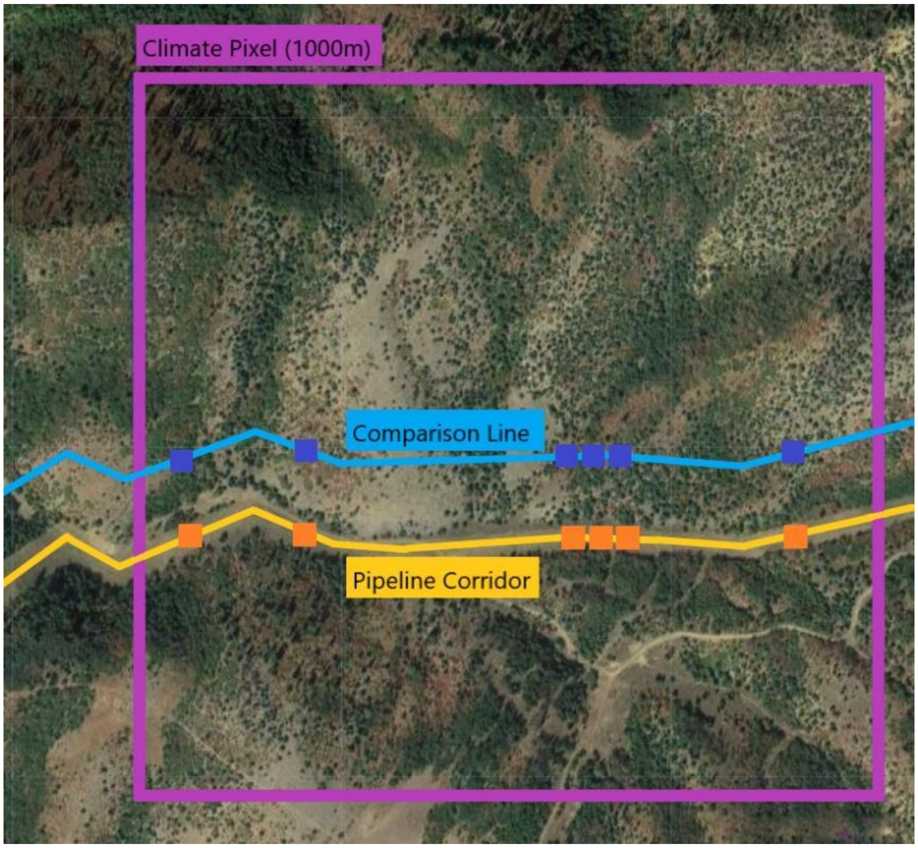

Fig. S2.

High resolution imagery of a pipeline corridor and the comparison line. These were used to limit data to pixels that represent corridor vegetation and identify undisturbed pixels on the comparison line that are likely similar in plant composition and productivity. All boxes represent true scale, with individual pixels along pipeline and comparison reference lines (shown here as shaded squares, 30 m^2) being averaged to the climate pixel level (1000 m^2).

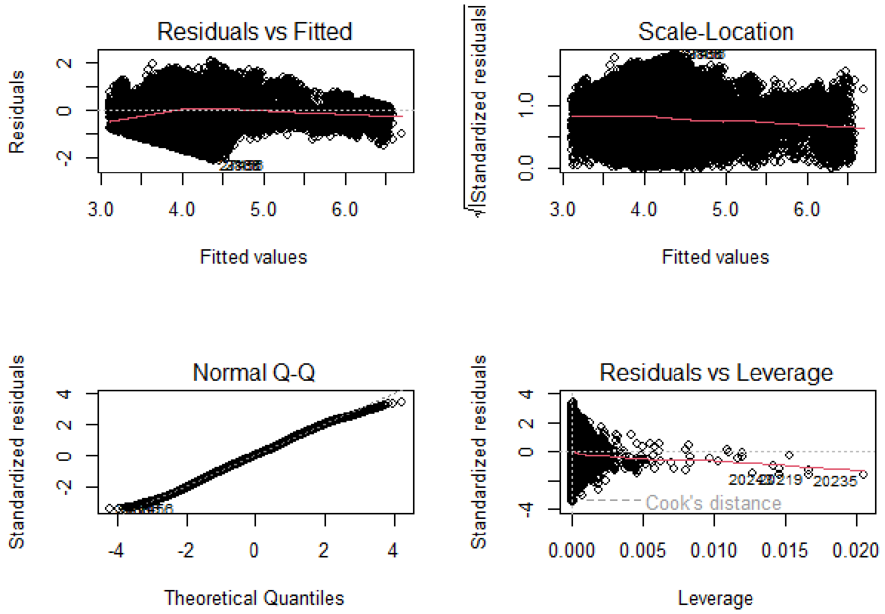

Fig. S3.

Diagnostic plots for the disturbance-only model.

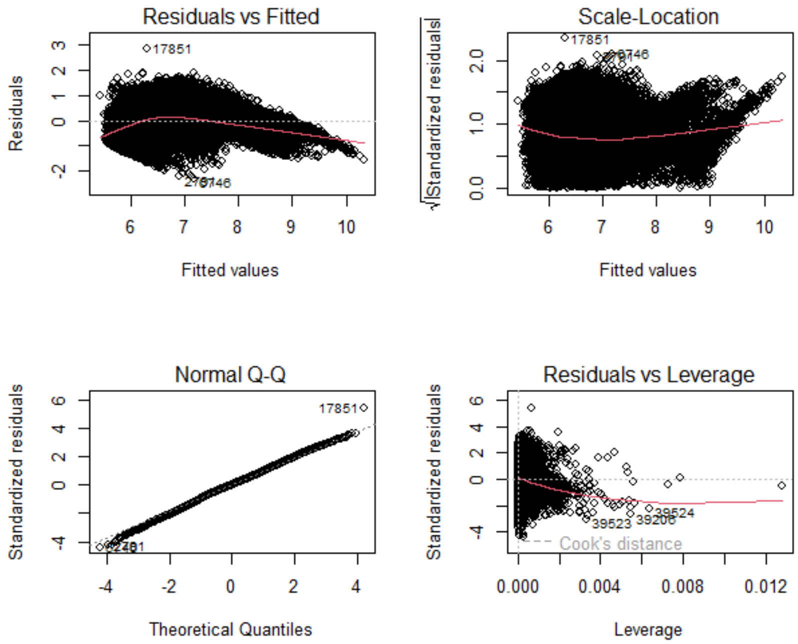

Fig. S4.

Diagnostic plots for the composition-only model.

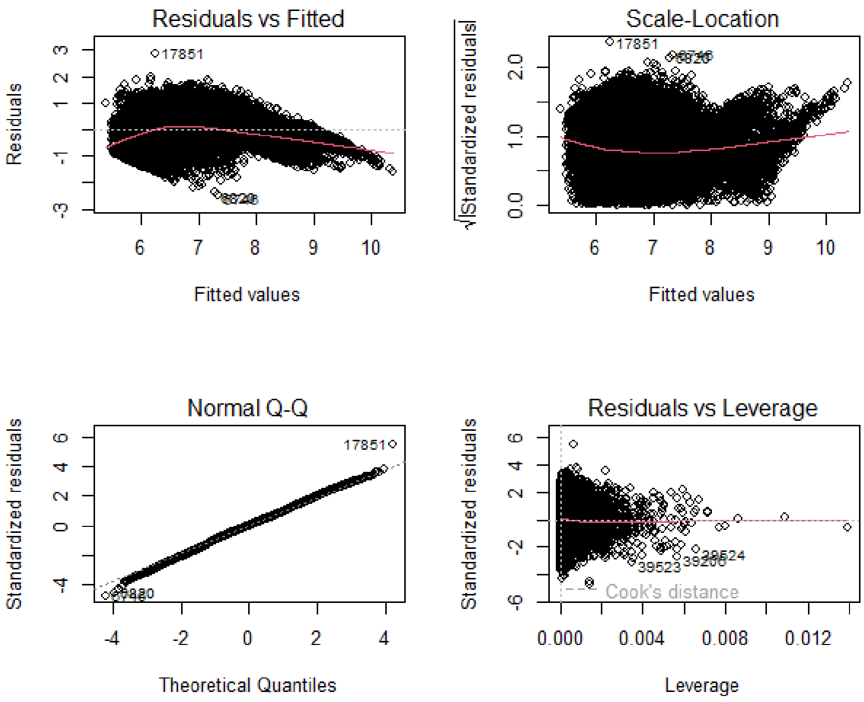

Fig. S5.
Diagnostic plots for the composition + disturbance model.

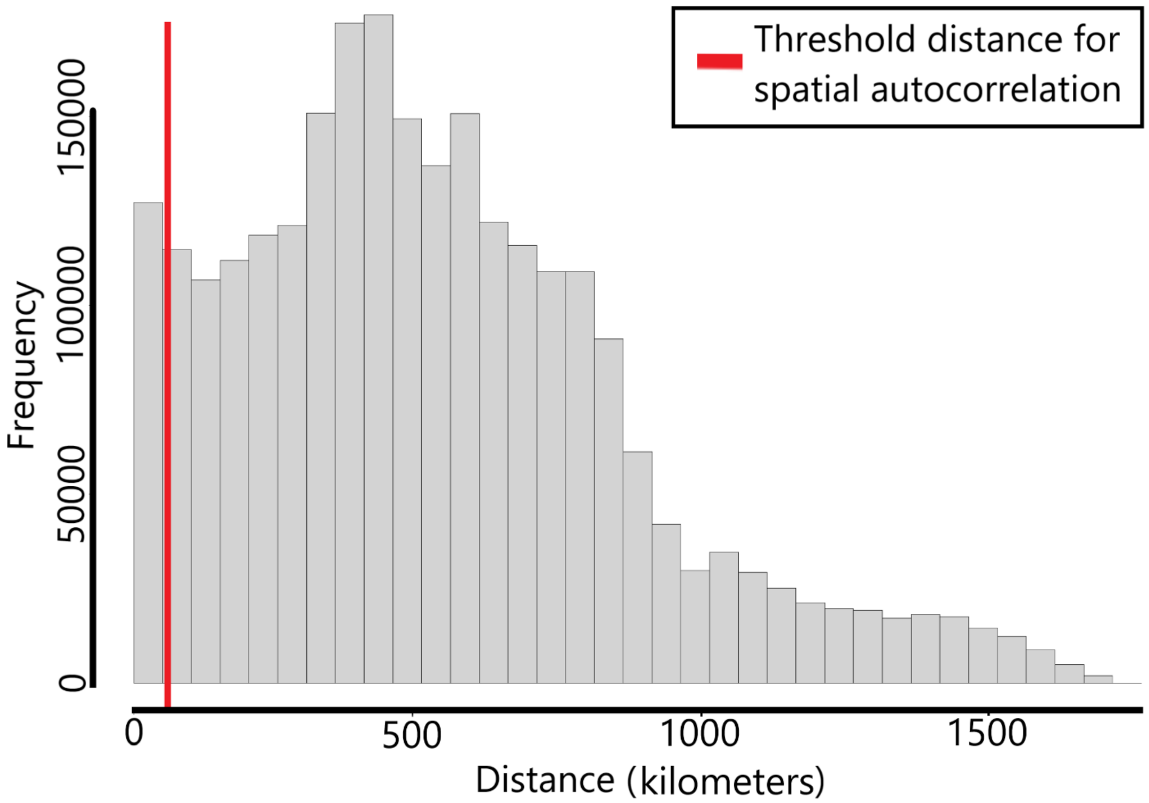

Fig. S6.

Frequency distribution of spatial distances between all pairs of data points. The red line indicates a minimum threshold distance beyond which spatial autocorrelation declines to zero in our disturbance-only model. All data pairs separated by distances greater than the red line can be considered independent.

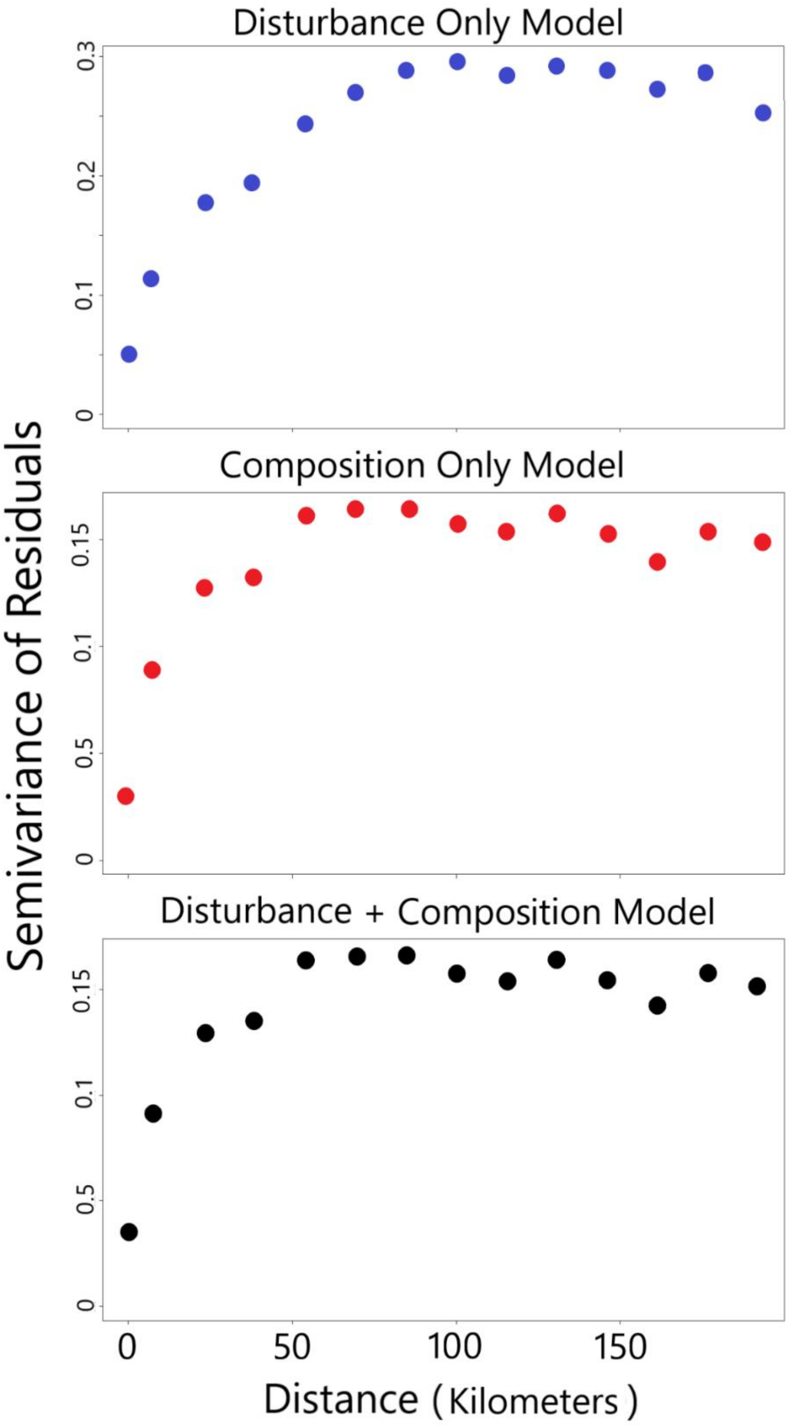

Fig. S7.

Semivariogram of the three models used in this study. Each point represents a pair of samples or observations. Y axis values indicate the semivariance of residuals from each model, a measure of the spatial dependency between two points. X axis represents distance between pairs of data.

Table S1.

Interpretation of coefficients from disturbance-only model (eq. 2).

Coefficient	Interpretation
α	Intercept
β_1	NPP response to average precipitation
β_2	Change in NPP with annual deviation in precipitation (Sensitivity)
β_3	Change in sensitivity across MAP
β_4	Disturbance effects on average NPP
β_5	Disturbance effects on sensitivity
β_6	This allows disturbance effects on sensitivity to vary across MAP
σ_p	Random effect of pipeline identity on main effect of disturbance
ε	Process error

REFERENCES AND NOTES

1. B. Poulter, D. Frank, P. Ciais, R. B. Myneni, N. Andela, J. Bi, G. Broquet, J. G. Canadell, F. Chevallier, Y. Y. Liu, S. W. Running, S. Sitch, G. R. Van Der Werf, Contribution of semi-arid ecosystems to interannual variability of the global carbon cycle. *Nature* **509**, 600–603 (2014).

2. A. Ahlström, M. R. Raupach, G. Schurgers, B. Smith, A. Arneth, M. Jung, M. Reichstein, J. G. Canadell, P. Friedlingstein, A. K. Jain, E. Kato, B. Poulter, S. Sitch, B. D. Stocker, N. Viovy, Y. P. Wang, A. Wiltshire, S. Zaehle, N. Zeng, The dominant role of semi-arid ecosystems in the trend and variability of the land CO_2 sink. *Science* **348**, 895–899 (2015).

3. T. E. Huxman, M. D. Smith, P. Fey, Convergence across biomes to a common rain-use efficiency. *Nature* **429**, 651–654 (2004).

4. G. E. Maurer, A. J. Hallmark, R. F. Brown, O. E. Sala, S. L. Collins, Sensitivity of primary production to precipitation across the United States. *Ecol. Lett.* **23**, 527–536 (2020).

5. A. J. Felton, R. K. Shriver, J. B. Bradford, K. N. Suding, B. W. Allred, P. B. Adler, Biotic vs abiotic controls on temporal sensitivity of primary production to precipitation across North American drylands. *New Phytol.* **231**, 2150–2161 (2021).

6. A. K. Knapp, M. D. Smith, Variation among biomes in temporal dynamics of aboveground primary production. *Science* **291**, 481–484 (2001).

7. Z. Hu, Q. Guo, S. Li, S. Piao, A. K. Knapp, P. Ciais, X. Li, G. Yu, Shifts in the dynamics of productivity signal ecosystem state transitions at the biome-scale. *Ecol. Lett.* **21**, 1457–1466 (2018).

8. A. Jentsch, P. White, A theory of pulse dynamics and disturbance in ecology. *Ecology* **100**, e02734 (2019).

9. B. W. Allred, W. K. Smith, D. Twidwell, J. H. Haggerty, S. W. Running, D. E. Naugle, S. D. Fuhlendorf, Ecosystem services lost to oil and gas in North America. *Science* **348**, 401–402 (2015).

10. E. J. Fusco, J. T. Finn, J. K. Balch, R. Chelsea Nagy, B. A. Bradley, Invasive grasses increase fire occurrence and frequency across US ecoregions. *Proc. Natl. Acad. Sci. U.S.A.* **116**, 23594–23599 (2019).

11. M. L. Brooks, B. Lair, "Ecological effects of vehicular routes in a desert ecosystem," United States Geological Survey, Las Vegas, NV, 2 March 2005.

12. A. G. Pendergrass, R. Knutti, F. Lehner, C. Deser, B. M. Sanderson, Precipitation variability increases in a warmer climate. *Sci. Rep.* **7**, 17966 (2017).

13. L. Wang, W. Jiao, N. MacBean, M. C. Rulli, S. Manzoni, G. Vico, P. D'Odorico, Dryland productivity under a changing climate. *Nat. Clim. Change* **12**, 981–994 (2022).

14. G. P. Asner, A. J. Elmore, L. P. Olander, R. E. Martin, A. T. Harris, Grazing systems, ecosystem responses, and global change. *Annu. Rev. Environ. Resour.* **29**, 261–299 (2004).

15. D. L. Hoover, B. Bestelmeyer, N. B. Grimm, T. E. Huxman, S. C. Reed, O. Sala, T. R. Seastedt, H. Wilmer, S. Ferrenberg, Traversing the wasteland: A framework for assessing ecological threats to drylands. *Bioscience* **70**, 35–47 (2020).

16. J. G. Pausas, J. E. Keeley, Wildfires and global change. *Front. Ecol. Environ.* **19**, 387–395 (2021).

17. G. Bridge, B. Özkaynak, E. Turhan, Energy infrastructure and the fate of the nation: Introduction to special issue. *Energy Res. Soc. Sci.* **41**, 1–11 (2018).

18. A. Bonet, Secondary succession of semi-arid Mediterranean old-fields in south-eastern Spain: Insights for conservation and restoration of degraded lands. *J. Arid Environ.* **56**, 213–233 (2004).

19. C. M. D’Antonio, P. M. Vitousek, Biological invasions by exotic grasses, the grass/fire cycle, and global change. *Annu. Rev. Ecol. Syst.* **23**, 63–87 (1992).

20. F. A. Bazzaz, The physiological ecology of plant succession. *Annu. Rev. Ecol. Syst.* **10**, 351–371 (1979).

21. M. Kleber, K. Eusterhues, M. Keiluweit, C. Mikutta, R. Mikutta, P. S. Nico, Mineral–organic associations: Formation, properties, and relevance in soil environments. *Adv. Agron.* **130**, 1–140 (2015).

22. L. Yahdjian, L. Gherardi, O. E. Sala, Nitrogen limitation in arid-subhumid ecosystems: A meta-analysis of fertilization studies. *J. Arid Environ.* **75**, 675–680 (2011).

23. J. Belnap, Surface disturbances: Their role in accelerating desertification. *Environ. Monit. Assess.* **37**, 39–57 (1995).

24. S. R. Abella, D. M. Gentilcore, L. P. Chiquoine, Resilience and alternative stable states after desert wildfires. *Ecol. Monogr.* **91**, e01432 (2021).

25. Z. Ratajczak, J. B. Nippert, S. L. Collins, Woody encroachment decreases diversity across North American grasslands and savannas. *Ecology* **93**, 697–703 (2012).

26. J. D. Corbin, C. M. D’Antonio, Competition between native perennial and exotic annual grasses: Implications for an historical invasion. *Ecology* **85**, 1273–1283 (2004).

27. T. Zhang, G. Yu, Z. Chen, Z. Hu, C. Jiao, M. Yang, Z. Fu, W. Zhang, L. Han, M. Fan, R. Zhang, Z. Sun, Y. Gao, W. Li, Patterns and controls of vegetation productivity and precipitation-use efficiency across Eurasian grasslands. *Sci. Total Environ.* **741**, 140204 (2020).

28. J. Fang, S. Piao, Z. Tang, C. Peng, W. Ji, Interannual variability in net primary production and precipitation. *Science* **293**, 1723–1723 (2001).

29. F. Zhang, J. A. Biederman, M. P. Dannenberg, D. Yan, S. C. Reed, W. K. Smith, Five decades of observed daily precipitation reveal longer and more variable drought events across much of the Western United States. *Geophys. Res. Lett.* **48**, 104550 (2021).

30. IPCC, Summary for policymakers, in *Climate Change 2022: Impacts, Adaptation, and Vulnerability Contribution of Working Group II to the Sixth Assessment Report of the Intergovernmental Panel on Climate Change*, H. O. Pörtner, D. C. Roberts, E. S. Poloczanska, K. Mintenbeck, M. Tignor, A. Alegría, M. Craig, S. Langsdorf, S. Löschke, V. Möller, A. Okem, Eds. (Cambridge Univ. Press, 2022), pp. 3–33.

31. N. Gorelick, M. Hancher, M. Dixon, S. Ilyushchenko, D. Thau, R. Moore, Google earth engine: Planetary-scale geospatial analysis for everyone. *Remote Sens. Environ.* **202**, 18–27 (2017).

32. United States Department of Transportation, *The National Pipeline Mapping System* (United States Department of Transportation, 2004).

33. N. P. Robinson, M.O. Jones, A. Moreno, T. A. Erickson, D. E. Naugle, B. W. Allred, Rangeland productivity partitioned to subpixel plant functional types. *Remote Sens.* **11**, 1427 (2019).

34. B. W. Allred, B. T. Bestelmeyer, C. S. Boyd, C. Brown, K. W. Davies, M. C. Duniway, L. M. Ellsworth, T. A. Erickson, S. D. Fuhlendorf, T. V. Griffiths, V. Jansen, M. O. Jones, J. Karl, A. Knight, J. D. Maestas, J. J. Maynard, S. E. McCord, D. E. Naugle, H. D. Starns, D. Twidwell, D. R. Uden, Improving Landsat predictions of rangeland fractional cover with multitask learning and uncertainty. *Methods Ecol. Evol.* **12**, 841–849 (2021).

35. M. Thornton, R. Shrestha, Y. Wei, P. Thornton, S. Kao, Wilson BE, *Daymet: Daily Surface Weather Data on a 1-km Grid for North America, Version 4* (ORNL DAAC, 2022).

36. R Core Team, *R: A Language and Environment for Statistical Computing* (R Foundation for Statistical Computing, 2022).

37. D. Bates, M. Mächler, B. Bolker, S. Walker, Fitting linear mixed-effects models using lme4. arXiv:1406.5823 [stat.CO] (2015).

38. A. Trabucco, Z. Robert, Global aridity index and potential evapotranspiration (ET0) climate database v3, figshare (2022); https://doi.org/10.6084/m9.figshare.7504448.v4.
